# Crystal structure of *P. falciparum* Cpn60 bound to ATP reveals an open dynamic conformation before substrate binding

**DOI:** 10.1038/s41598-021-85197-3

**Published:** 2021-03-15

**Authors:** Brian Nguyen, Rui Ma, Wai Kwan Tang, Dashuang Shi, Niraj H. Tolia

**Affiliations:** Host-Pathogen Interactions and Structural Vaccinology Section, Laboratory of Malaria Immunology and Vaccinology, Division of Intramural Research, National Institute of Allergy and Infectious Disease, National Institutes of Health, Rm 4NN08, Building 29B, 9000 Rockville Pike, Bethesda, MD 20892 USA

**Keywords:** Chaperones, X-ray crystallography, Parasitology

## Abstract

*Plasmodium falciparum* harbors group 1 and group 2 chaperonin systems to mediate the folding of cellular proteins in different cellular locations. Two distinct group 1 chaperonins operate in the organelles of mitochondria and apicoplasts, while group 2 chaperonins function in the cytosol. No structural information has been reported for any chaperonin from plasmodium. In this study, we describe the crystal structure of a double heptameric ring *Plasmodium falciparum* mitochondrial chaperonin 60 (Cpn60) bound with ATP, which differs significantly from any known crystal structure of chaperonin 60. The structure likely represents a unique intermediate state during conformational conversion from the closed state to the opened state. Three of the seven apical domains are highly dynamic while the equatorial domains form a stable ring. The structure implies large movements of the apical domain in the solution play a role in nucleotide-dependent regulation of substrate binding and folding. A unique 26–27 residue insertion in the equatorial domain of *Plasmodium falciparum* mitochondrial chaperonin greatly increases both inter-ring and intra-ring subunit–subunit interactions. The present structure provides new insights into the mechanism of Cpn60 in chaperonin assembly and function.

## Introduction

Group 1 and group 2 chaperonins are two distinct classes of chaperones that exist in diverse organisms to assist with protein folding in an ATP-dependent manner^1,2^. Group 1 chaperonins are found in the cytosol of bacteria, such as GroEL from *Escherichia coli* or Cpn60 from *Thermus Thermophilus,* while in eukaryotic organisms, they are located in the chloroplast or mitochondria^3^. Group 2 chaperonins include the archaeal thermosome, the eukaryote tailless complex polypeptide 1 (CCT), and the eukaryotic tailless complex polypeptide 1 ring complex (TRiC)^4^. In both chaperone groups, each monomer is comprised of three domains: an equatorial domain, an intermediate domain, and an apical domain. Seven or eight monomers assemble together to form a heptameric or octameric ring, and two rings interact back-to-back through the equatorial domains to form tetradecameric or hexadecameric double-ring structures^4^.

**Figure 1:**
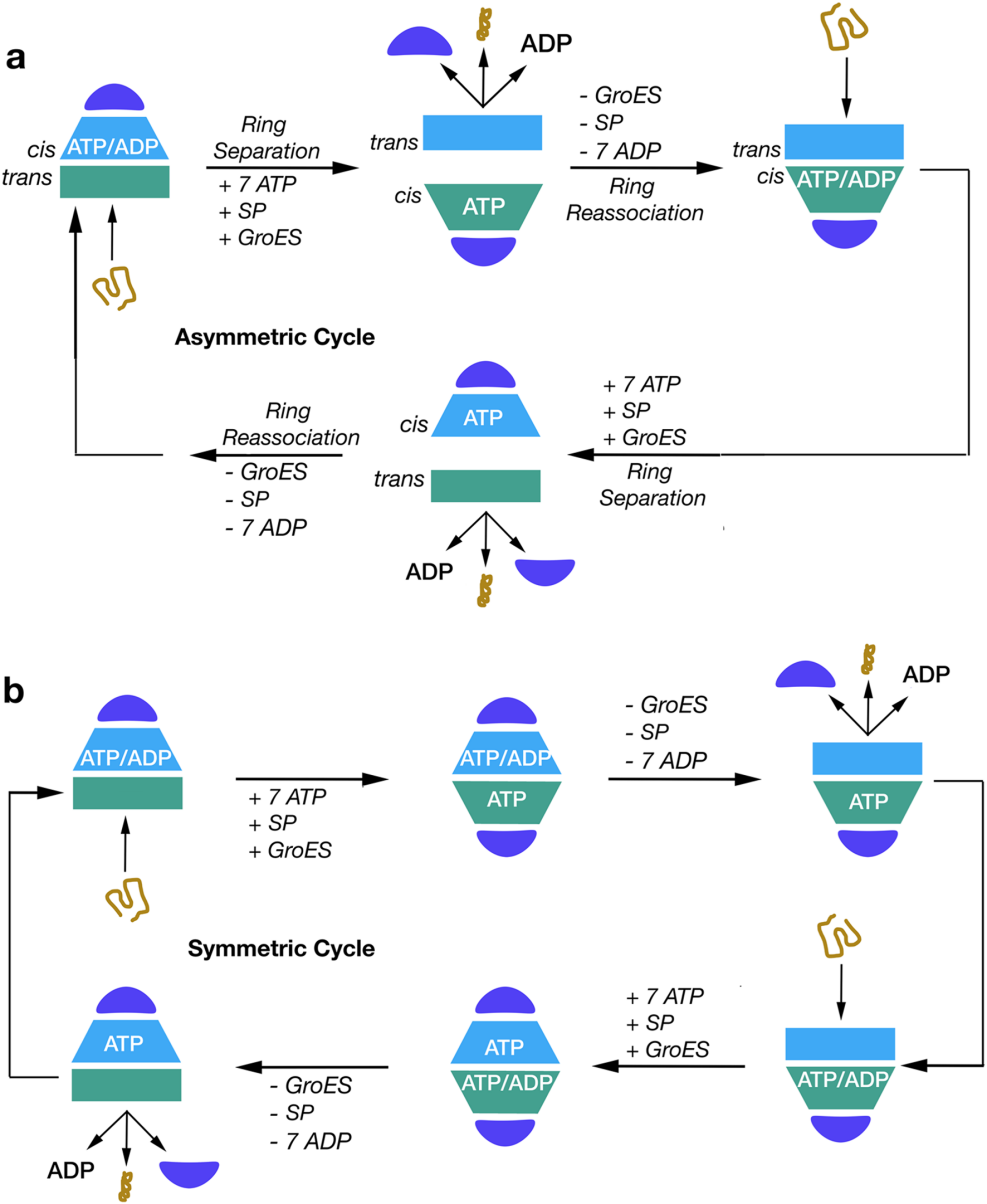
Reaction mechanism of GroEL/GroES. (**a**) Asymmetric cycle of GroEL. Adapted and modified from Hayer-Hartl et al.^1^ and Yan et al.^16^. (**b**) Symmetric cycle of GroEL. Adapted and modified from Lizuka and Funatsu^10^. Violet semi-circle represents a Cpn10 heptamer, blue or green rectangles/trapezoids represent the closed/open conformation of two distinct Cpn60 heptamers, tan elongated/condensed line represents unfolded or folded SP.

Most of the group 1 chaperonins are homo-oligomers. In contrast, the hexadecamer of group 2 chaperonins are composed of eight different monomers despite having similar three-dimensional structures^2^. The equatorial domains contain the ATP binding site, the intermediate domain serves as a hinge connecting the equatorial to the apical domain, and the apical domain is involved in substrate binding. Group 1 chaperonins require the binding of a co-chaperone Cpn10 to the apical domain to encapsulate the substrate protein (SP), while the apical domain of group 2 chaperonins utilizes a built-in lid. The binding of Cpn10 to group 1 chaperonins is critical for the folding mechanism that forms bullet-shaped (Cpn60_14_Cpn10_7_), American football-shaped (Cpn60_14_Cpn10_14_), or American half-football-shaped (Cpn60_7_Cpn10_7_) complexes at distinct stages of the folding mechanism (Fig. 1).

Since the first GroEL structure from *E. coli*^5^, multiple structures of group 1 chaperonin from 6 different organisms have been deposited into the Protein Data Bank (PDB) (Supplementary Table S1). A vast majority of structures are from *E. coli,* making *E. coli* GroEL and its partner GroES the best-characterized group 1 chaperonin. GroEL consists of two heptameric rings of identical monomers, each with a molecular weight of approximately 60 kDa (also termed Cpn60 or Hsp60 in other organisms). The partner co-chaperonin, GroES, is a dome-shaped heptameric ring of identical monomers with a monomeric molecular weight of approximately 10 kDa (also termed Cpn10 or Hsp10 in other organisms). GroEL requires ATP binding in order to shift from a closed conformational state to an open conformational state that allows for SP and GroES binding^6^. In this open conformation, the intermediate and apical domains of GroEL rotate and move upward to form a complex with GroES. Two cycles have been suggested for the function of GroEL. The prevailing asymmetric cycle which (Fig. 1a) states that only one heptameric ring can partake in folding at a time^6–9^ and the symmetric cycle^10–15^ (Fig. 1b) where both rings can fold simultaneously. Recent research has also determined another mechanism of GroEL within the asymmetric cycle, inter-ring separation, where ATP hydrolysis and binding causes disassociation between the two heptameric rings^16^.

However, there is evidence that chaperonins from organisms other than *E. coli* may have slightly different mechanisms. It is therefore imperative to study group 1 chaperonins from diverse organisms to holistically define the mechanism of chaperonin action. For example, the structure determination of a human mitochondrial chaperonin demonstrated that a novel intermediate exists with subunit asymmetry within the rings (different conformations for intra-ring subunits) and nucleotide symmetry between the rings (ATP or ADP binds both rings)^17^. Furthermore, the human chaperonin forms football-shaped complexes with a different inter-ring arrangement from that of GroEL^17^. Additionally, the crystal structure of the football complex with both rings bound to ADP suggests that human Cpn60 follows a symmetrical cycle, where both rings can undergo hydrolysis at the same time. Cryo-electron microscopy recently revealed that in addition to the full football-shaped Cpn60_14_Cpn10_14_ (PDB: 6MRC), the half-football-shaped complex consisting of Cpn60_7_Cpn10_7_ (PDB: 6MRD) can independently exist in solution, with both forms active in assisting the folding of imported mitochondrial proteins^18^; this suggests ring separation may be a possible step during the chaperonin cycle^19^. Similarly, the bullet-shaped structure from *T. thermophilus* exhibited a significant deviation from the sevenfold symmetry for the apical domain around the *cis*-cavity^20^. Studies on the chaperonin from *Chlamydomonas* chloroplast demonstrated a significantly different mechanism for chaperonin to capture, encapsulate, fold, and release substrate proteins. The binding and hydrolysis of ATP induce not only both positive (intra-ring) and negative (inter-ring) cooperative actions, but also promotes the partial disassembly of chaperonin into monomer, a phenomenon unique to chloroplast chaperonin^21,22^. Research on Cpn60 from *Paracocus denitrificans* indicated that the inter-ring interaction in this Cpn60 is weakened compared to *E. coli* GroEL, thus both single ring and double ring forms were observed^23^. In summary, due to structural and functional variations between Cpn60 orthologs, studies on Cpn60 from diverse organisms are warranted to better define the overall mechanism of Cpn60.

Malaria remains a major public health problem worldwide resulting in nearly half a million deaths and 200 million clinic illnesses annually^24^. Cpn60 is a ubiquitous protein that exists in nearly all domains of life including bacteriophage^25^ and plays an essential role in maintaining protein homeostasis^26^. In yeast, deletion of mitochondrial Cpn60 is lethal^27^. In mice, the inactivation of its homolog causes embryonic mortality^28^. Heat-shock proteins such as Cpn60 are often upregulated in response to stressful conditions. PfCpn60 must cope with two radically different host environments, mosquito and human, making it an interesting ortholog to study. In order to understand its mechanism in *P. falciparum*, we report the first crystal structure of *P. falciparum* Cpn60 (PfCpn60) bound with ATP, which reveals the large conformational dynamic of the apical domain relative to the intermediate and equatorial domains.

## Results

### Negative stain electron microscopy of WT-PfCpn60 and D474A-PfCpn60

Both group 1 and 2 chaperonins can be identified in the genome of the malaria parasite *P. falciparum*. Two group 1 Cpn60 genes exist, with one located on chromosome 12 that expresses a Cpn60 believed to localize to mitochondria^29,30^, and a second located on chromosome 10 with an encoded Cpn60 that is expected to function specifically inside the apicoplast^30,31^. Compared to known structures of Cpn60, the mitochondria-specific *P. falciparum* Cpn60 contains insertions including a 26–27 residue insert in the equatorial domain and a 10 residue insert in the apical domain (Supplementary Fig. S1), as well as *N*- and *C*-terminal extensions.

**Figure 2:**
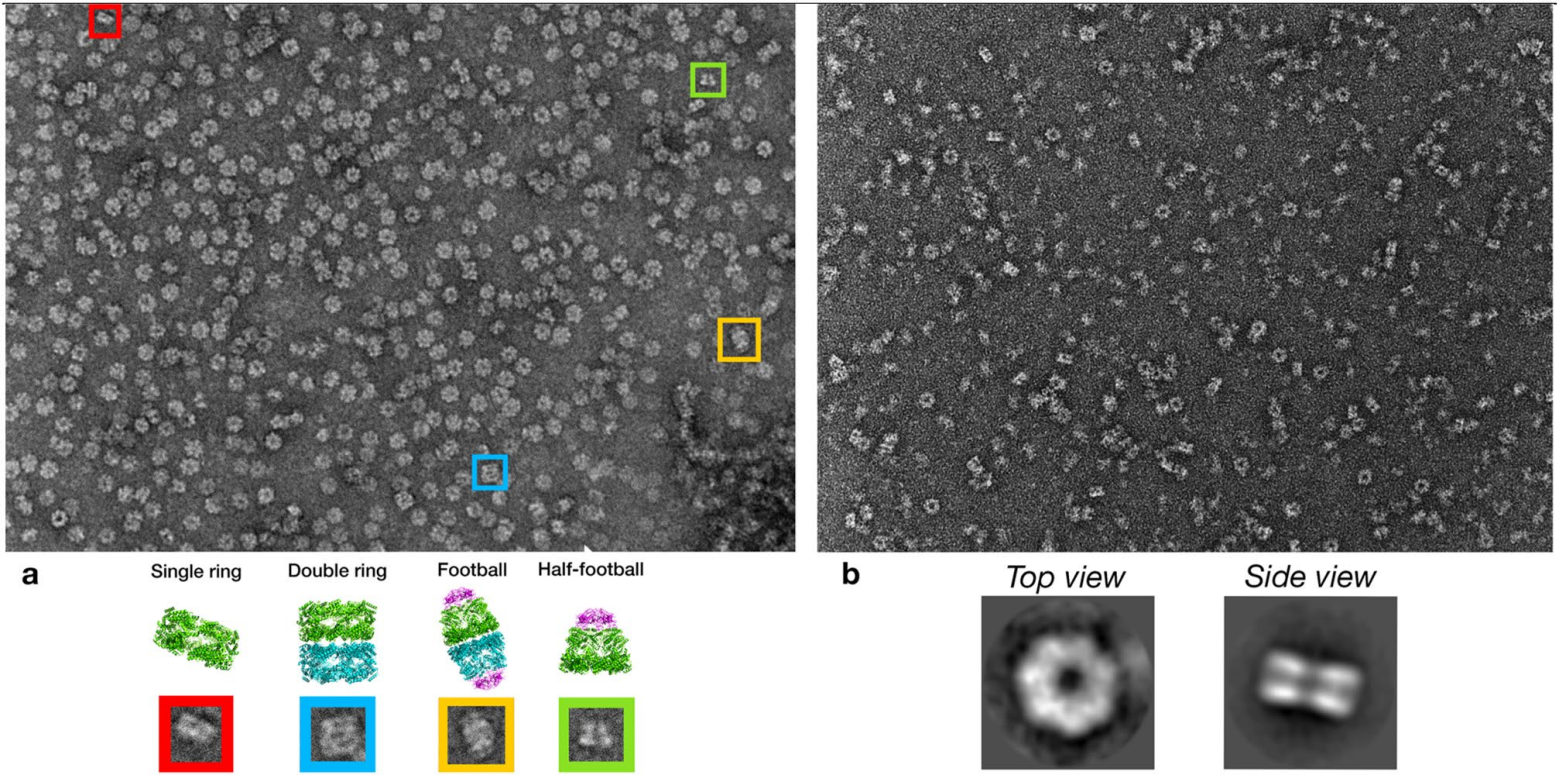
Negative stain electron microscopy analysis of PfCpn60. (**a**) Wild-type Cpn60 co-purified with Cpn10 with 1 mM ATP. Bottom panel shows the represented view of single ring, double ring, football and half-football structures of WT-Cpn60. (**b**) ATP hydrolysis deficient D474A mutant co-purified with Cpn10 with 1 mM ATP. Bottom panel represents the 2D classification of the negative stains, demonstrating the top view and side view of D474A PfCpn60 mutant, which is in a single ring conformation.

After nickel-his affinity and size-exclusion chromatography purification, both WT-PfCpn60 and ATP hydrolysis-deficient mutant D472A-PfCpn60 were submitted to negative stain electron microscopy. In the WT-PfCpn60 sample, several forms of structures including single-ring, double-ring, half-football, and whole football coexist (Fig. 2a). In contrast, most structures in the D474A-PfCpn60 sample are single-ring structures (Fig. 2b). Consistent with this observation, crystals were grown only from the D474A-PfCpn60 sample. No crystals were grown from WT-PfCpn60 probably due to the structural heterogeneity caused by the fast hydrolysis of ATP in WT-PfCpn60 which occurs in the matter of seconds^14^. Comparatively, it is estimated that the D474A mutation slows hydrolysis to a rate of around 40 min. Since only the D474A-PfCpn60 crystal structure was determined, the following results and discussion of the PfCpn60 structure is based on D474A-PfCpn60.

### Structure determination of D474A-PfCpn60

Matthew’s coefficient calculation estimated that there are seven subunits in an asymmetric unit in the crystal structure of PfCpn60, with a coefficient of 3.9 and an estimated solvent content of 68%^32^, a significantly higher solvent content than most other proteins due to the large cavity inside the chaperonin structure. A blast search of the PDB database indicated the closest model of PfCpn60 was the Cpn60 structure from *T. thermophilus* (TtCpn60, PDB: 4V4O) with a sequence identity of 39%^20^. The structure of 4V4O is a bullet-shaped complex, Cpn60_14_Cpn10_7_, with one heptameric ring in closed conformation with no nucleotide bound, and the other heptameric ring in opened conformation with ADP and Cpn10 binding^20^. Since PfCpn60 was co-purified with PfCpn10, the opened conformation of TtCpn60 was used as the search model first. However, no molecular replacement solution was found.

Interestingly, no molecular replacement solution was found using the closed conformation of TtCpn60 as well, implying the conformation of PfCpn60 in the current structure may differ from both conformations of TtCpn60 in 4V4O. We hypothesized that the flexibility within the intermediate hinge region may have caused the positions of the equatorial and apical domains to differ from the search model. Therefore, in order to identify the molecular replacement solution, the individual domains were used as the search model. The molecular replacement solution was identified using the equatorial domain alone from both conformations of the TtCpn60 structure. Furthermore, the equatorial domain from the closed conformation ring performed better as a search model than the opened domain, indicating that the current structure is closer to the closed conformation. Careful inspection of molecular packing for the molecular replacement solution indicated that enough empty space was available for the packing of the intermediate plus apical domains, but not enough for the additional Cpn10 binding to the apical domain, suggesting that Cpn10 may not be present in the current structure. Eventually, the molecular replacement solution was successfully found by searching one copy of the heptamer of the equatorial domain alone and seven copies of the intermediate plus apical domain. The final solution gave a total likelihood gain of 7360.

Inspection of the molecular replacement solution indicated that Cpn60 is well-packed in the crystal structure, confirming the absence of Cpn10. A reason for Cpn10 not co-crystallizing with Cpn60 may be due to weak interaction between Cpn10 and Cpn60 resulting in the loss of Cpn10 during purification, due to specific conditions during crystallization, or due to the ATP binding state. We performed a negative stain electron microscopy analysis which revealed that the major species existing in solution is a heptamer single ring structure of Cpn60 without the presence of Cpn10 (Fig. 2b), even though both Cpn60 and Cpn10 were present on the SDS-PAGE gel following gel filtration purification (Supplementary Fig. S2). In addition, the low pH of 4.2 in the crystallization buffer may have further prevented the formation of the Cpn10 and Cpn60 complex. Finally, the eventual shift from open to closed conformation by ATP hydrolysis may have hampered Cpn10 binding and retention.

**Figure 3:**
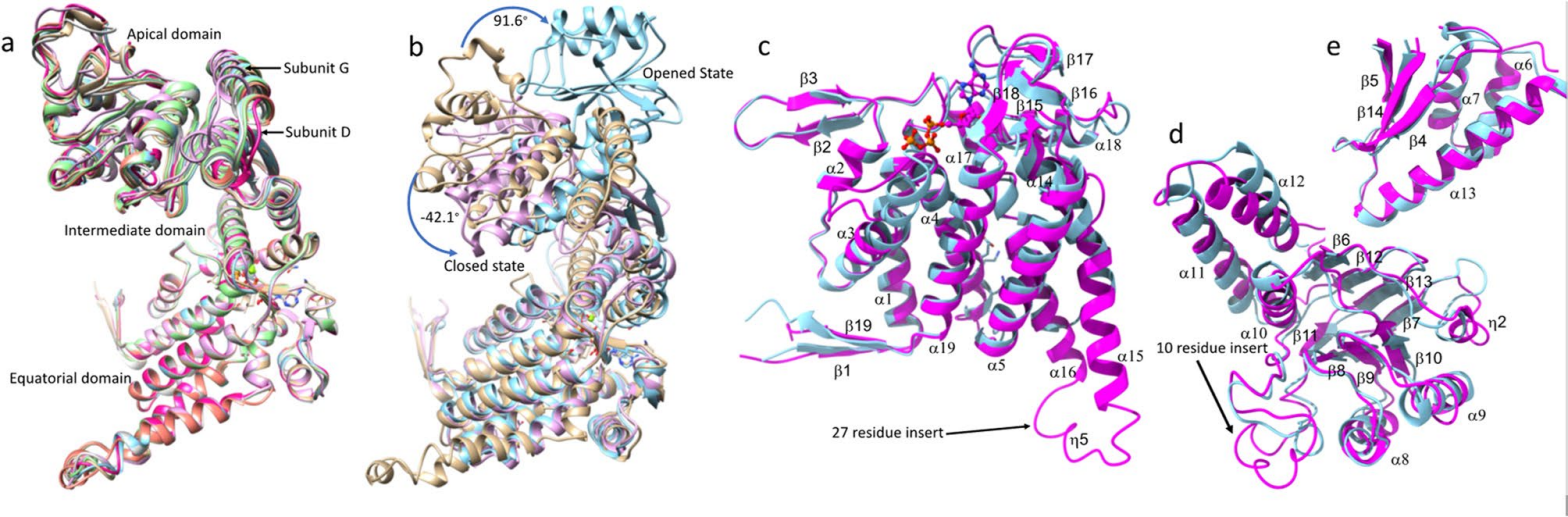
Structure of PfCpn60 and comparison with TtCpn60. (**a**) Superimposition of seven subunits, shown in different color ribbons, in the asymmetric unit using equatorial domain showing the variations of the apical and intermediate domains. Subunit G (shown in purple ribbons) is in the most closed conformation while subunit D (shown in red ribbons) is in the most opened conformation. (**b**) The conformational differences of PfCpn60 (subunit A, shown in brown ribbons) with the opened conformation of TtCpn60 (4V4O, subunit A, shown in light-blue ribbons) and the closed conformation (4V4O, subunit H, shown in pink ribbons). The superimposition was carried on using their equatorial domain. (**c**) Superimposition of PfCpn60 (subunit A, shown in magenta ribbons) and TtCpn60 (closed form shown in light-blue ribbons) for the equatorial domain only. The 27-residue insert is highlighted. (**d**) Superimposition of PfCpn60 (shown in magenta ribbons) and TtCpn60 (closed form shown in light-blue ribbons) for the apical domain only. (**e**) Superimposition of PfCpn60 (shown in magenta ribbons) and TtCpn60 (closed form shown in light-blue ribbons) for the intermediate domain only. The 10-residue insert is highlighted.

One heptameric ring was found per asymmetric unit of the PfCpn60 structure. The sevenfold NCS axis was almost parallel with the crystallographic *C*-axis. A second heptameric ring in an adjacent asymmetric unit that is related by twofold crystallographic symmetry results in a tetradecameric complex with the rings stacked back-to-back, as observed in the other Cpn60 structures. The seven subunits in the asymmetric unit are in slightly different conformations and differ from the closed and opened conformations typically observed for other Cpn60 molecules (Fig. 3a,b, Table 1 and Supplementary Table S2). The apical domain in PfCpn60 has a relative angle movement of as large as − 42° and 92° compared to the closed and opened conformations (subunit A and subunit H in 4V4O), respectively, which were calculated by first superimposing the equatorial domain as the reference, then determining the angle of rotation required to superimpose the apical domain using the secondary structure superimpose tool in COOT (Fig. 3b). This large movement explained why the search models from both closed and opened conformations were unsuccessful in molecular replacement. A search on the PDBeFold server^33^ (https://www.ebi.ac.uk/ssm) with the final refined structure revealed that the current PfCpn60 structure is closest to that of ATP-bound EcGroEL structure (PDB ID: 2C7E)^34^, with a root mean square deviation (RMSD) of 2.3 Å.

**Table 1:** Relative rotation angle (°) of apical and intermediate domains.

Subunit	Apical domain	Intermediate domain
A	0	0
B	0.7^a^	1.2
C	1.3^a^	1.3
D	2.4	1.4
E	1.9	1.4
F	1.1^a^	1.8
G	− 7.3	− 1.0

^a^The electron density of apical domains in subunit B, C and F is weak. Thus, the values are not reliable.

The electron density in the equatorial domain is of very high quality with strong backbone density and clearly identifiable side chains. In particular, the electron density for the 26–27 extra amino residues between K509 and E536, compared to *E. coli* and *T. thermophilus* Cpn60 sequence (Supplementary Fig. S1), is traceable and a model can be built (Supplementary Fig. S3). The diverse conformations and flexibility of the apical domain conformations hampered structure determination of these segments due to poor electron density, particularly in the apical domains of subunit B, C and F. However, the electron density of the apical domains of subunits A, D, E, and G allowed for structural modeling, particularly with chain A having good electron density and model-building properties.

The flexibility observed in the PfCpn60 structure may have functional significance in the binding of Cpn10 and substrates. Although the electron density of the apical domains for subunit B, C, and F are weak, the significant residual electron density in their corresponding regions indicated their presence and dynamic nature. In order to maintain the completeness of the model, the models of the apical domains in subunits B, C, and F were created through NCS operation of the apical domain of subunit A as a template.

### Structure of *P. falciparum* mitochondrial Cpn60

**Figure 4:**
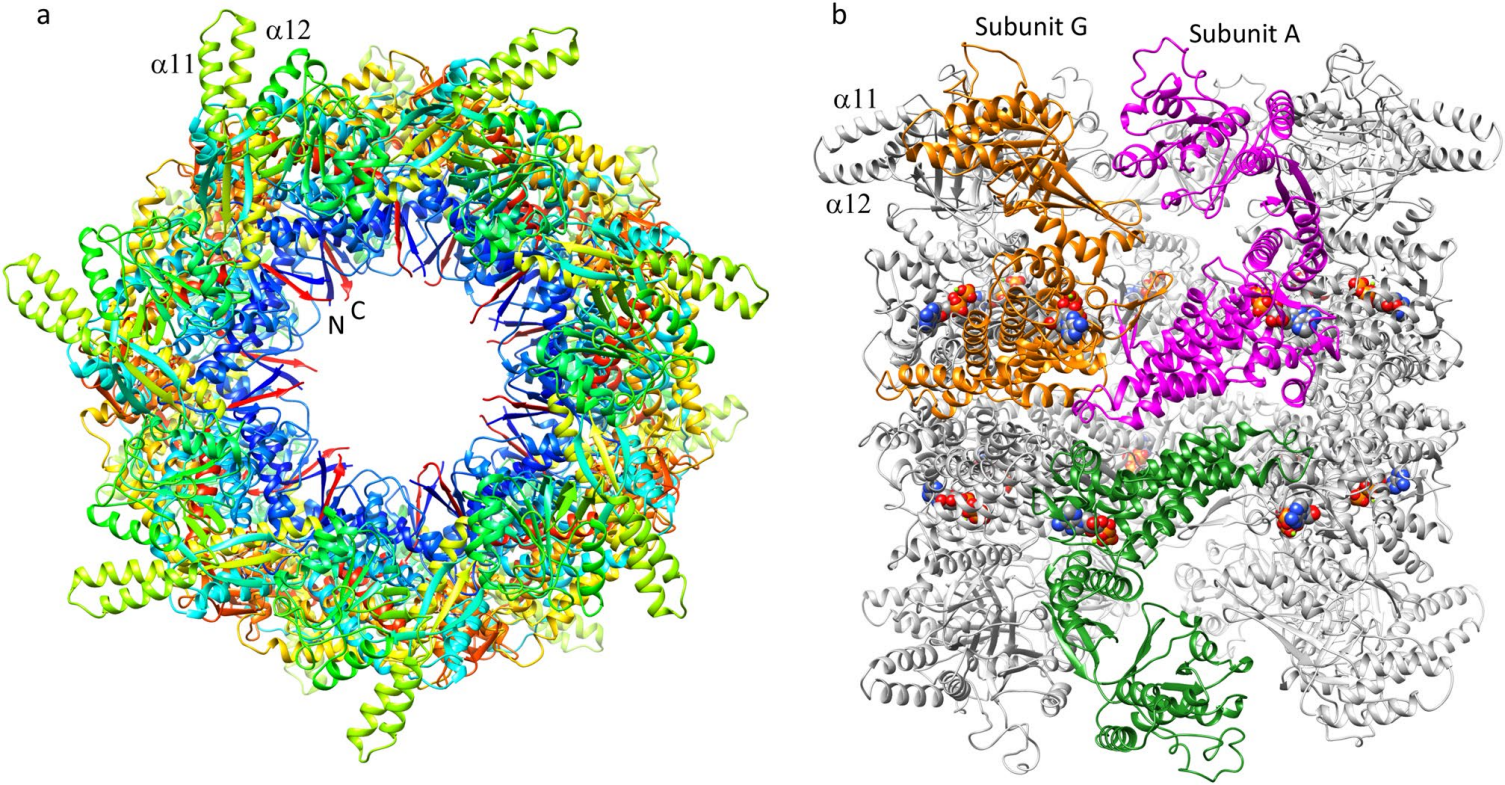
Structure of PfCpn60 oligomer. (**a**) The top view of PfCpn60 tetradecamer. The protein was shown in rainbow ribbon colored from blue (*N*-termini) to red (*C*-termini). Two termini form an anti-parallel β-strand arrangement towards to the inner cavity. (**b**) The side-view of PfCpn60 tetradecamer. The top heptameric ring interacts with the bottom heptameric ring in a back-to-back manner and are related to each other by twofold crystallographic symmetry. The protein is shown in ribbon with subunits G and A in orange and magenta, respectively. The interacting subunit in the opposite ring is shown in forest green. Bound ATP is shown as filled spheres.

The overall structure of PfCpn60 represents a typical group 1 chaperonin, with a sevenfold symmetrical cylinder consisting of two back-to-back stacked rings composed of seven subunits (Fig. 4a,b). The *N*- and *C*-termini are angled towards the inner cavity while the α11–α12 loop of the apical domain is located on the outermost surface of the cylinder. The two rings were exactly related to each other by the two-fold crystallographic symmetry. Similar to the structures of other Cpn60 orthologs, each subunit consisted of an equatorial, intermediate, and apical domain (Fig. 3a).

The equatorial domain consists of 11 α-helices in the core and four anti-parallel two-stranded β sheets on the surface. The ATP binding-site is located on the edge of the equatorial domain with a phosphate group binding to the *N*-terminal end of α4 helix, and adenosine and sugar groups surrounded by β16, α17, β18, and β15–α14, α2–β2 loops (Fig. 3C). The intermediate domain is composed of three α helix bundle (α6, α7, and α13) and one three-stranded β sheets (β4↑β14↑β5↓) (Fig. 3E). The apical domain is formed by double layers of β-sheets, which consist of one four-stranded β-sheet (β11↓β8↑β9↑β10↑) and one four-stranded antiparallel β-sheet (β6↓β12↑β13↓β7↑), as a core with a three α helix bundle (α10, α11, and α12) hanging on one side of the edge and other three helices (α8, α9 and η2) on the other side of the edge (Fig. 3d). In the open conformation of TtCpn60, the residues from equivalent helix α8 and α9 interact with residues from the mobile loop of Cpn10^20^.

Although structures of diverse Cpn60 orthologs with double heptameric rings have been determined (Supplementary Table S1), a vast majority of them are closed-conformations of apo-Cpn60 without ATP or ADP bound. ATP-bound symmetric double-ring structures have only been determined at low resolution by cryo-electron microscopy^34,35^. No crystal structure for an ATP-bound double-ring lidless complex (Cpn60_14_) of group 1 chaperonin currently exists, possibly due to the high flexibility of the apical domain in this conformation.

There are several unique features in the current PfCpn60 structure. In contrast to most other known Cpn60 structures, which are either in opened or closed conformation, the present PfCpn60 structure represents a unique ATP-bound conformation. The superimposition of the current structure with the closed and opened conformation of TtCpn60 (using the equatorial domain) confirmed that the conformation of the PfCpn60 structure is a unique ATP-induced conformation distinct from both the closed and open conformation (Fig. 3b). The whole PfCpn60 structure cannot be superimposed to either opened or closed conformation complexes, but each domain can be superimposed with those of the closed conformation of TtCpn60, with rsmd of 1.0, 1.1, and 2.0 Å for equatorial, intermediate, and apical domains, respectively (Fig. 3c–e).

In comparison with the sequences of other Cpn60 orthologs with known structures (Supplementary Fig. S1), PfCpn60 has two long insertions: an extra 10 amino residues between L378 and N387, and an extra 27 amino residues between K510 and S536. The residues L378–N387, which form an extended loop between helix α10 and β11, are located at the edge of the apical domain close to helix α8, which is involved in the binding of co-chaperone Cpn10 (Fig. 3d). The residues E513–E538 form two extended α-helices (α15 and α16) and a loop with a short η5 helix to interact with an adjacent subunit in the intra-ring (Fig. 4a). Furthermore, the extended α-helices are part of an inter-ring interface and may enhance inter-ring interactions (Fig. 4b).

### Intra-ring subunit–subunit interactions

**Figure 5:**
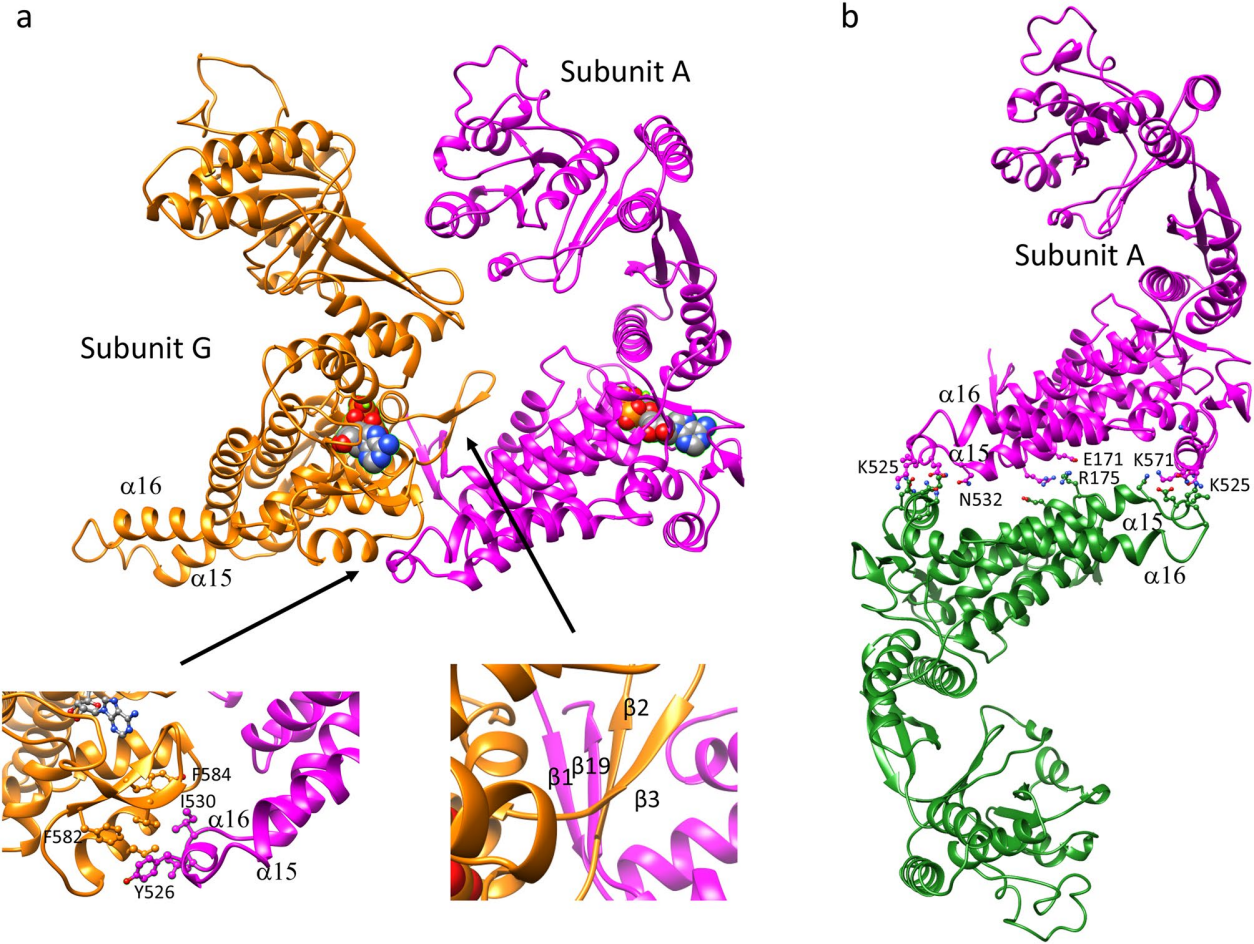
Intra-ring (**a**) and inter-ring (**b**) subunit–subunit interactions. Subunit A is shown in magenta ribbon while the adjacent subunit G is shown in orange ribbon. The opposite subunit in the opposite ring is shown in forest-green ribbon. ATP is shown as filled spheres. The selected side chains involved in the interaction are shown in balls-sticks.

The intra-ring subunit-subunit interactions consist of two parts: inter-equatorial domain interactions, and intermediate domain and apical domain interaction (Fig. 5a). The interactions among intermediate and apical domains vary with conformational change, but the interactions between equatorial domains are consistent across all monomers. One general feature for intra-ring subunit-subunit interactions is that an antiparallel β-loop (residues 103–117, β2↑β3↓) projects from the body of the equatorial domain towards the inner surface of its right-handed adjacent subunit, where it forms a parallel β-sheet structure with its *C*-terminal segment (residues 624–629, β19↑). This *C*-terminal segment further interacts with the *N*-terminal segment (residues 70–74, β1↓) in an anti-parallel β-strand arrangement to form a four-stranded β-sheet (β1↓β19↑β2↑β3↓) that glues the two adjacent subunits together (Fig. 5a). The four-stranded β-sheet interaction across the intra-ring adjacent subunits is likely to be essential for Cpn60 to form a heptameric ring since this feature is conserved in all Cpn60 structures in different conformational forms (1KP8, 1PCQ, and 5OPX) and from different organisms (4V4O, 1IOK, and 6MRC). Deletion mutagenesis of the *C*-terminal segment of EcGroEL impaired the EcGroEL assembly supporting this notion^36^.

One unique feature of PfCpn60 that impacts intra-ring subunit-subunit interactions is the extra 27 residue insertion (residues 513–539, α15–α16) that forms an extended α-helix loop and contacts the left-handed intra-ring subunit (Fig. 5a). This results in a much larger intra-ring subunit-subunit interaction area of 1828 Å^2^ in PfCpn60 as compared to other Cpn60 structures (Table 2). Specifically, residues Y526, L529, and I530 from the extended α-helix loop make hydrophobic interactions with residues I572, F582, Y584, and F591 from adjacent intra-ring subunits (Fig. 5a).

**Table 2:** Comparison of subunit-subunit interactions among different Cpn60s.

PDB	Intra-ring buried area (Å^2^)	Inter-ring buried area (Å^2^)		
		1	2	3
This study	1828	683.0	62.8	55.2
1KP8	1486	180.9	131.7	
1OEL	1542	189.1	188.0	
1PCQ_cis_	1581	198.4	197.8	
1PCQ_trans_	1557			
5OPX	1697	197.1	0.0	
1IOK	1193	198.5	161.4	
5DA8	1284	234.8	152.1	
5CDI	1442	209.0	167.8	
6MRC	1808	165.1	73.9	

### Inter-ring subunit-subunit interactions

As revealed in the other Cpn60 structures, the two heptameric rings contact each other primarily at two sites with two different opposite ring subunits, termed left and right sites in the equatorial domain^5^. In the bullet-shaped EcGroEL structure (PDB: 1PCQ), two salt-bridges, R452-E461 and van der Waals contacts among S463, V 464, and N467, form the right site contact. The van der Waals contacts among K105, A108, and A109 in the *cis*-ring, and A109, G110, and M111 in the *trans*-ring, contribute to the left site interactions. However, these residues are not conserved in PfCpn60 and the specific inter-ring interactions are not directly comparable (Supplementary Fig. S1).

PDBePISA web server^37^ was used to analyze the inter-ring subunit–subunit interaction and the results are listed in Table 2. The right site contacts of Cpn60 are better retained across different conformations than the left site contacts due to the relative movement of inter-ring subunits. This relative movement was calculated by superimposing the equatorial domain of one subunit in one of the heptameric rings, then determining the angle of rotation required to superimpose the equatorial domain of the subunit in the opposite heptamer ring. With the structure of PfCpn60 subunit A as the reference, the relative rotation angles are listed in Table 3 for Cpn60 from different organisms. Nucleotide binding (1OEL vs. 1KP8) caused a slight relative equatorial domain movement (< 1°). The formation of a bullet-shaped complex induced about 7° of the relative movement; as a result, the interactions of the left inter-ring contacts became weaker (1PCQ vs. 1OEL). The formation of football-shaped complexes (5OPX and 6MRC) causes a larger relative movement of ~ 20° (5OPX vs. 1OEL) for inter-ring subunits and weakens the left site interaction. Different forms of structures, which are caused by the binding of different nucleotides and substrate protein, appear to affect inter-ring interactions significantly.

**Table 3:** Relative movement of inter-ring subunit of other Cpn60 to PfCpn60.

PDB	Inter-ring relative rotation angle (**°**)	Composition
1KP8	6	EcCpn60_14_AGS_14_
1OEL	6	EcCpn60_14_
1PCQ	14	EcCpn60_14_Cpn10_7_ADP_7_
5OPX	26	EcCpn60_14_Cpn10_14_ADP_14_
1IOK	8	PdCpn60_14_
5DA8	7	CtCpn60_14_
5CDI	4	CrCpn60_14_
6MRC	29	HsCpn60_14_Cpn10_14_ADP_14_

The inter-ring subunit-subunit interface buried area of 683 Å^2^ in PfCpn60 is much larger than the buried area of ~ 200 Å^2^ in other Cpn60 structures, due to the extra 27 residue insertion in PfCpn60 (Table 2). Approximately 18–24 residues from both rings form the inter-ring interface. Specifically, five hydrogen bonding interactions of R175–E171, K571–E528, K577–E528, K571–N532, and K525–N575 and two salt bridges of R175–E171 and K577–E528 may be involved in forming this interface (Figs. 4a, 5b). L529 N565, N568, K571, N575 interact with residues K528, L529, N532 from an adjacent inter-ring subunit. In comparison to the EcGroEL structure with bound ATP analog (1KP8), a 6° relative angle movement was observed in the current PfCpn60 structure (Table 3), calculated by superimposing the equatorial domain of subunit A as a reference, then determining the angle of rotation required to superimpose the equatorial domain of the opposing subunit using the secondary structure superimpose tool in COOT (Fig. 5b). Different inter-ring interactions in PfCpn60 may affect ring association-disassociation and inter-ring cooperativity.

### The ATP binding site of Cpn60

**Figure 6:**
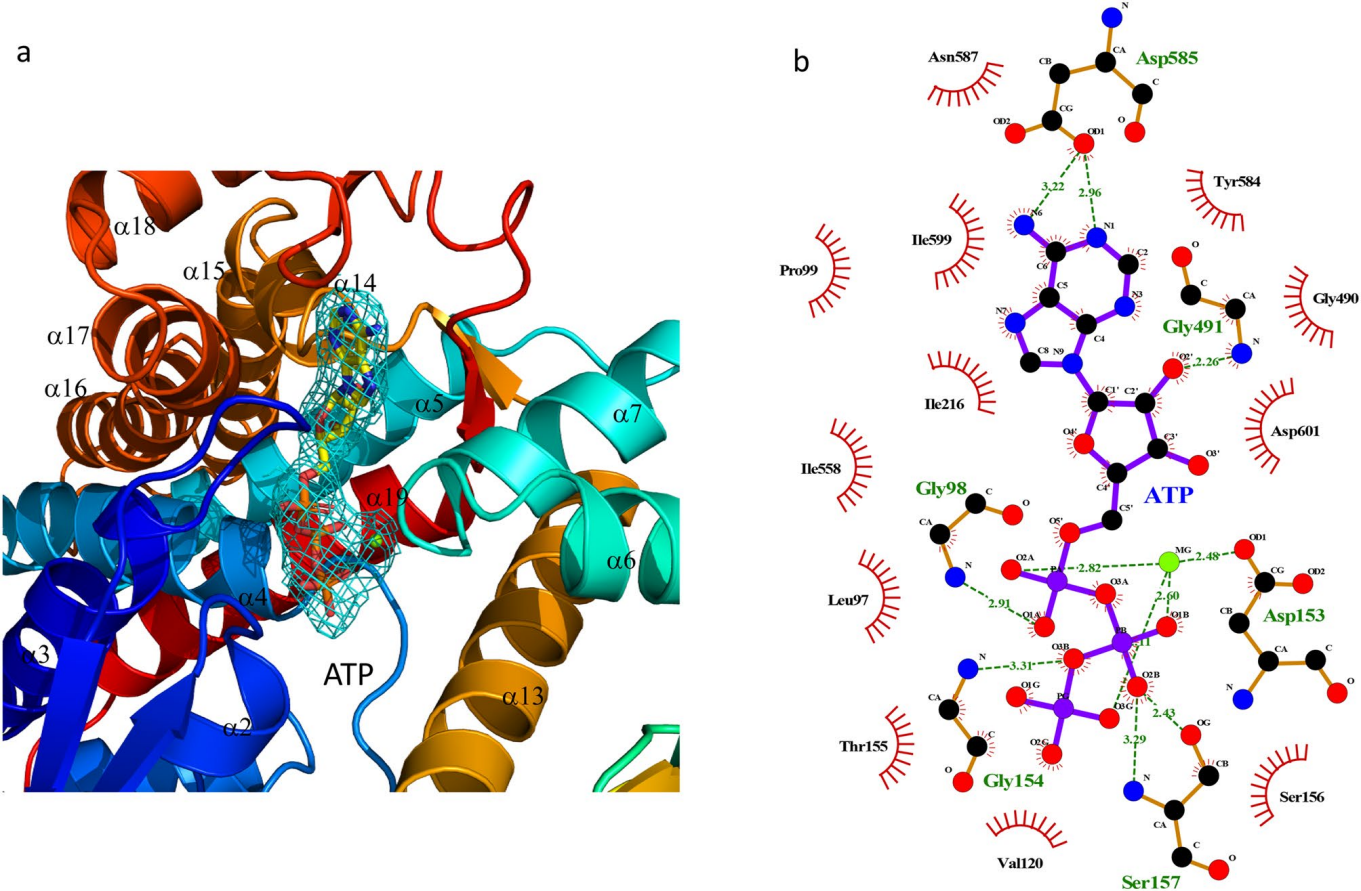
ATP binding site of PfCpn60. (**a**) The electron density map (2*F*_*o*_–*F*_*c*_) around bound ATP and Mg^2+^ with ATP are shown as ball-and-sticks, and the electron density is shown in sky-blue mesh cage. The figure was generated using Pymol. (**b**) The interactions of ATP with surrounding residues. The ATP and Mg^2+^ are shown as ball-and-sticks. Hydrogen bonds are shown as green dotted lines, while the spoked arcs represent protein residues making nonbonded contacts with the ligand. The figure was made using Ligplot^+^^38^.

Inspection of the electron density indicated that the ATP is present in the conserved binding site (Fig. 6a and Supplementary Fig. S4b). ATP binds to PfCpn60 in a similar fashion as observed in EcGroEL (PDB: 2C7E and 1KP9) and TtCpn60 (PDB:4V4O). The side chains of D153, S156, and mainchain N of G154 are involved in hydrogen bonding to the phosphate group, the side chains of D691 are involved in hydrogen bonding to the sugar group, and sidechains of I599, P99, V586, I216, and I558 are involved in hydrophobic interaction to the adenosine ring. The hydrogen bond interactions of the side chains of N587 and D585 help anchor the position of the adenosine group (Fig. 6b). The hydrogen bond interactions of the side chains of N587 and D585 also help anchor the position of the adenosine group. The phosphate group binding motif, A151–G152–D153–G154–T155, is absolutely conserved across known Cpn60 (Supplementary Fig. S1). Although the sequence of PfCpn60 aligns well with the sequences of other group 1 chaperonins, the residues between T144 and G154 between helix α3 and α4, the loop structure of PfCpn60 in which part of *C*-terminal ⍺3 helix melts, is significantly different from other group 1 chaperonins (Supplementary Fig. S5). The flexibility of this loop appears to be important for the entry of ATP and the exit of ADP.

### Highly dynamic apical domains

**Figure 7:**
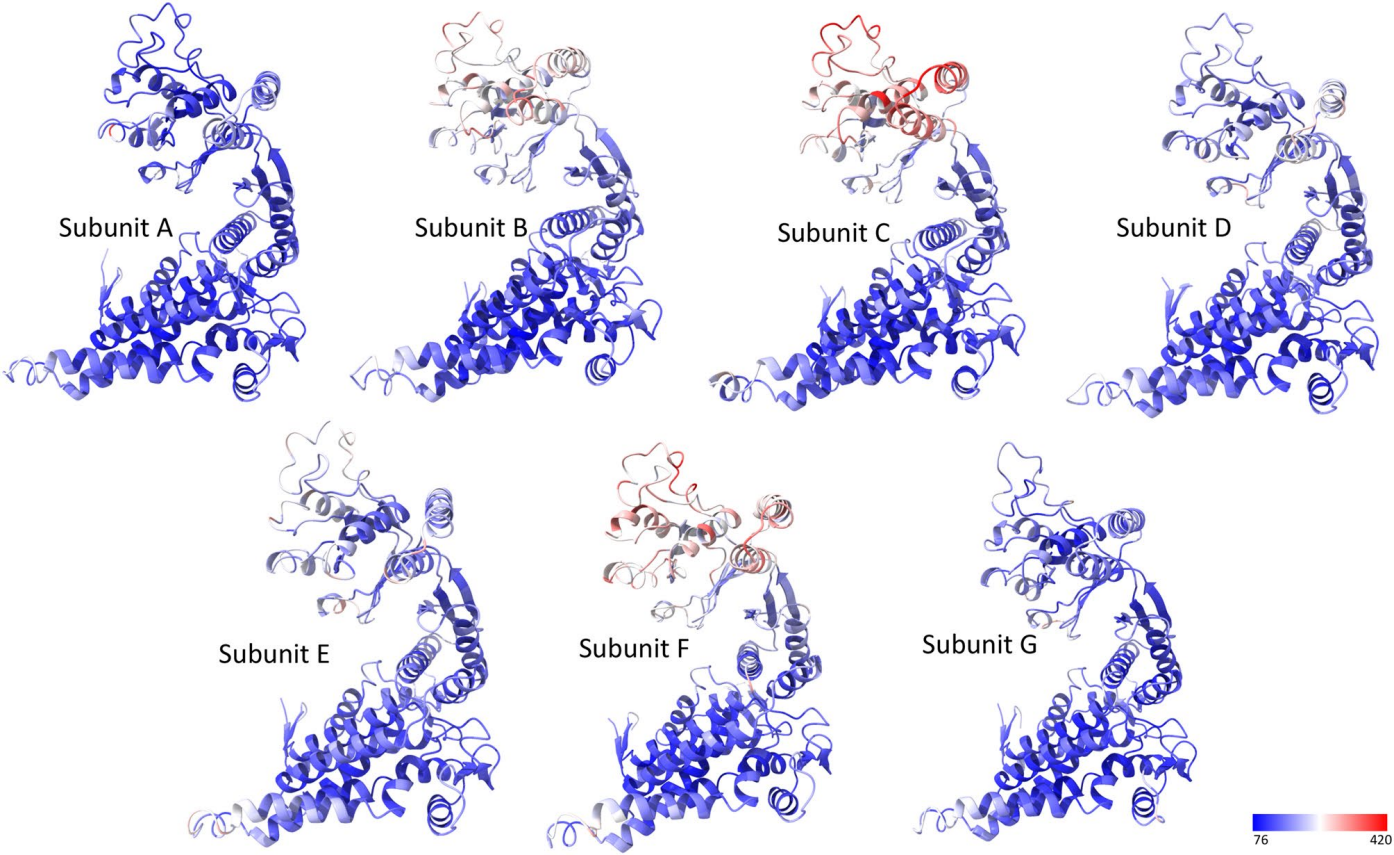
Dynamic of the equatorial, the intermediate and the apical domains for individual subunit. The ribbons are colored according to the *B* factors from low (blue) to high (red).

The overall average *B* factor of the model was 163.5 Å^2^. In the model, the equatorial domain had a relatively low *B* factor of 132.5 Å^2^, while the intermediate and apical domains had higher *B* factors of 155.8 and 206.7 Å^2^, respectively, reflecting the different mobility of different domains with the lowest being the equatorial domain and the highest being the apical domain (Table 4). This phenomenon has been observed in many other Cpn60 structures^23,39^. Furthermore, it was interesting to observe that the temperature factors of the equatorial domains were similar for each intra-ring subunit, while the temperature factors of the intermediate and apical domains were very different for each intra-ring subunit (Fig. 7, Supplementary Table S3). Inspection of the molecular packing in the crystal revealed that a strong correlation exists between the high-temperature factor and the amount of crystal lattice constraints. This correlation implies that the intermediate domain, particularly the apical domain of PfCpn60, is extremely mobile in the solution and can be ordered by packing in the crystal. In the current structure, the apical domains of subunit B, C, and F are much more mobile (weak electron density and high-temperature factors) than those of subunit A, D, E, and G, due to less interaction with other symmetry-related molecules. Crystallographic constraints may explain why apical domains in different conformations were observed in individual intra-ring subunits in many other group 1 chaperonin structures^17,20,40,41^. The conformational variation for each subunit may also reflect chaperonin’s functional requirement for the chaperonin to bind various protein substrates to facilitate their folding, rather than a single protein.

**Table 4:** Data collection and refinement statistics.

Data collection	
Space group	P622
**Cell dimensions**	
a, b, c (Å)	281.77, 281.77, 299.32
Resolution (Å)	63.0–3.69 (3.79–3.69)^a^
Total reflections	667,450
Unique reflections	74,184 (5,368)
R_meas_	0.133 (1.50)
I/σI	12.9 (1.7)
Completeness (%)	98.8 (98.3)
CC_1/2_	99.9 (60.7)
Redundancy	9.1 (8.1)
Wilson *B* factor (Å^2^)	117.9

Refinement	
Resolution (Å)	48.2–3.69 (3.80–3.69)
No. of reflections	74,077 (7262)
R_work_/R_free_	0.227/0.277
**No. of atoms**	30,938
Protein	30,714
Ligand/ion	224
**B-factors (Å**^**2**^**)**	
Protein	163.5
Equatorial domain	132.5
Intermediate domain	155.8
Apical domain	206.7
ATP	126.5
**rmsd**	
Bond lengths (Å)	0.003
Bond angles (°)	0.803
**Ramachandran plot (%)**	
Favored	92.60
Outliers	0.0

^a^Values in parentheses are for highest-resolution shell.

## Discussion

This structure is the first group 1 chaperonin structure from *P. falciparum*, the most prevalent parasite on the African continent responsible for nearly half a million deaths and 200 million clinical illnesses each year^24^. The availability of the PfCpn60 structure provides a new group 1 chaperonin from a different organism that will help to better understand the overall function and mechanism of group 1 chaperonins. EcGroEL is the best-characterized chaperonin and several different forms of structures have been determined (Supplementary Table S1): (1) Apo or ADP or phosphothiophosphoric acid adenylate ester (AGS) bound double-ring Cpn60_14_ closed form (1OEL, 4KI8, 1KP8)^40–42^; (2) ATP bound lidless double-ring Cpn60_14_ partially opened form (2C7E and 4AAQ)^34,35^; (3) ADP or ATP bound single lid bullet-shaped form, Cpn60_14_Cpn10_7_ (1PCQ), with ADP or ATP binding only to the *cis* ring^43^; (4) ATP or ADP + Be_3_F bound double lidded football-shaped opened form, Cpn60_14_Cpn10_14_ (3WVL and 4PKO), with ADP + Be_3_F or ATP binding to both rings^44,45^. The PfCpn60 D474A mutant crystal structure revealed a symmetric double-ring complex with all subunits occupied by ATP that is similar to pre-open ATP-bound GroEL Cryo-EM structures 4AAU and 4AB2^35^. Negative stain images reveal varying forms of PfCpn60 for the wildtype (Fig. 2a) compared to the single ring form seen in the D474A mutant (Fig. 2b). It is likely that the slowed hydrolysis in the mutant staggers the particles into a similar stage and allows for the crystallization of intermediate conformations. Based on conformational studies on GroEL by Clare et al., this pre-open ATP-bound conformation occurs between the closed and open conformations, to prepare for SP and Cpn10 binding^35^. The most apparent feature shared among pre-open conformation structures is the outward position of α11 and α12 and an inward shift of the intermediate domain^35^.

**Figure 8:**
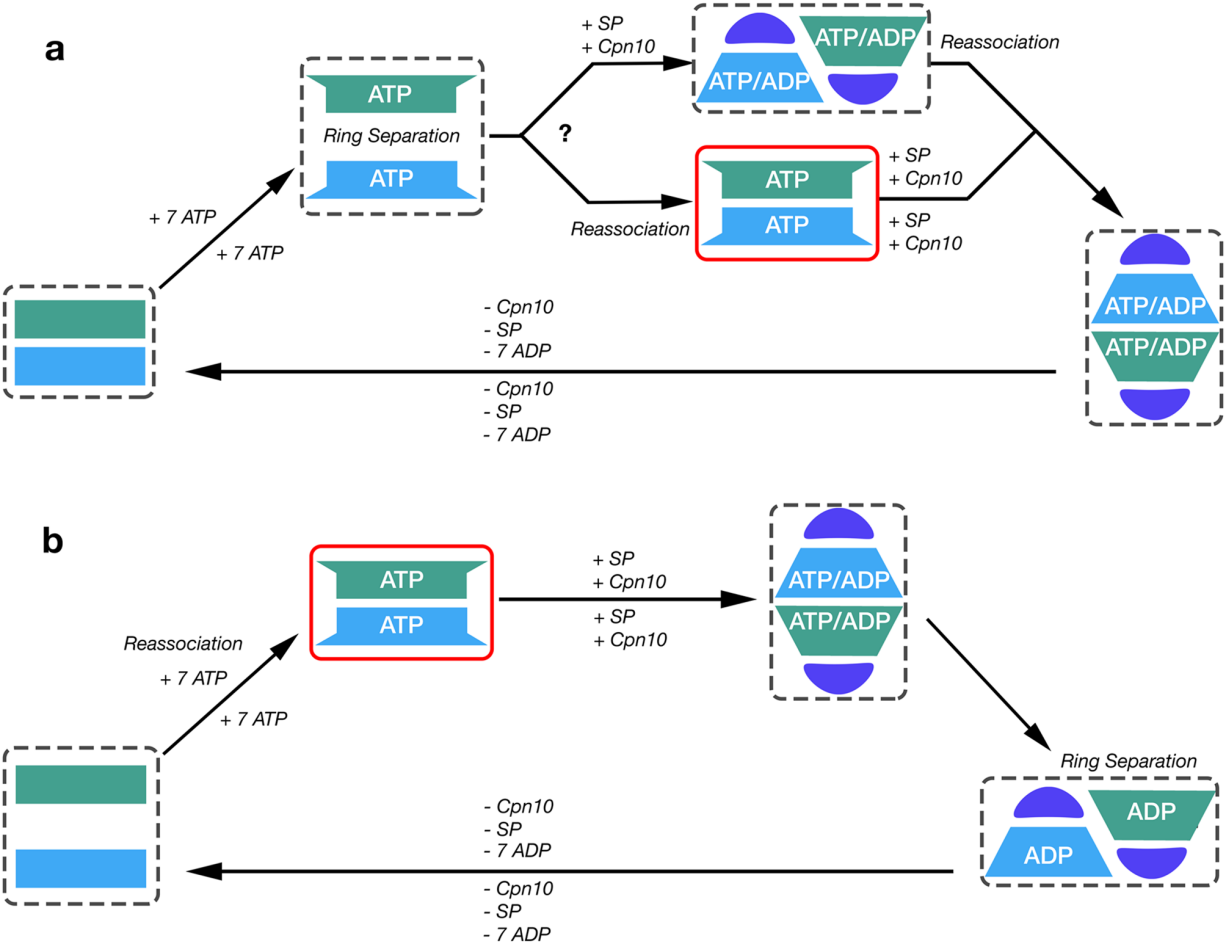
Possible models of the PfCpn60/Cpn10 reaction cycle, based on previous research, incorporating the crystal structure (solid red box) and the possible states observed with negative stain electron microscopy in Fig. 2 (dashed red box). Schematic drawing shows a simplified reaction cycle indicating ATP-bound as a distinct state during the conversion from apo-closed state to Cpn10 + SP + ATP-bound opened state. In addition, the ring dissociation due to ATP binding is shown with model a demonstrating the mechanism adapted from Yan et al.^16^. and model b demonstrating the mechanism adapted from Gomez-Llorente et al.^18^. Violet semi-circle represents a Cpn10 heptamer, blue or green rectangles/trapezoids represent the closed/open conformation of two distinct Cpn60 heptamers, tan elongated/condensed line represents unfolded or folded SP.

In the presence of Cpn10 and ATP, Cpn60 from diverse organisms can be found to exist as in both single- and double-ring forms^18,23^. Furthermore, it was found that the single-ring assembly is enough for productive chaperonin-mediated protein folding^18,46^ and human Cpn60 (also termed Hsp60) does not display any negative inter-ring cooperativity at any point of the reaction cycle. PfCpn60 has been observed in the single ring conformation (Fig. 2b) suggesting the occurrence of inter-ring separation. This study is consistent with the two prevailing models of Cpn60 action^18,47^, and the pre-open conformation described here can be readily placed in both cycles (Fig. 8).

As in all other Cpn60 structures, both *N*- and *C*-termini are oriented inward towards the inner cavity. The structure is based on PfCpn60 sequence (XP_001350715) from 69 to 631 only. After cleavage of the first 23 residues of the mitochondrial signal peptide, predicted based on the SignalP 3.0 Server^48^, the mature PfCpn60 should have 46 extra residues at the *N*-terminus before G69, and 87 extra residues at the *C*-terminus after E631. It is interesting to notice that there are 51 negatively charged residues of Asp + Glu at its *C*-terminus. The extra residues protruding from the equatorial domain towards the inner cavity in the wild-type PfCpn60 certainly block free passage between the cavities in both rings. The negative charges inside the equatorial ring in wild-type PfCpn60 may also prevent double-ring formation. Further study of PfCpn60 in the native state may be essential to fully understand the structure and mechanism of PfCpn60.

Although PfCpn60 was co-purified with Cpn10, it is interesting to note that Cpn60 did not crystallize with Cpn10. The dissociation of Cpn10 from Cpn60 should not be due to the crystallization buffer because negative stain electron microscopy did not observe the presence of Cpn10-bound complexes comprising the football or bullet. The interactions between Cpn10 and Cpn60 are mainly through the interactions of the residues from the mobile loop of Cpn10 and the residues from α8 and α9 of Cpn60, which include the critical triplet residues of the mobile loop^16,18,45^. The triplet residues in PfCpn10 and L31-F32-L33 are comparable to the corresponding triplets I31-M32-L33 in human mitochondrial Cpn10 and I25-V26-L27 in GroES^30^. The corresponding interacting residues such as L303, E304, and L328 in Cpn60 are also conserved (Supplementary Fig. S1). Therefore, in the presence of ATP and Cpn10, PfCpn60 should likely form a football-shaped or half-football-shaped complex with Cpn10. The absence of Cpn10 in the current structure is likely due to the poor binding to Cpn60 in the ATP-bound open form in the absence of SP.

The tetradecameric assembly of PfCpn60 is mainly held together by the subunit-subunit interaction of the equatorial domains. The apical domain is highly dynamic; its position is affected easily by nucleotide binding, protein substrate binding, and potentially the crystal packing environment. Asymmetry with subunits in a differing apical domain conformation has been observed in several Cpn60 crystal structures^17,20^. It is plausible that this asymmetric phenomenon may reflect the extreme conformational flexibility of the Cpn60 apical domain in solution. Examples of subunit asymmetry are well-demonstrated by human Cpn60 structures, which were recently determined by both X-ray crystallography and single-particle cryo-EM (PDB: 4PJ1 and 6MRC). Both structures are American football-shaped Cpn60_14_Cpn10_10_ structures bound with ADP. In the X-ray crystallographic structure (4PJ1), the apical domains in two of the subunits (subunit G in one ring and subunit N in the opposite ring) are very different from the conformations of the apical domains in other intra-ring subunits, exhibiting as large as 100° of counterclockwise rigid body movement of the apical domain compared to those in other subunits^17^. However, in the structure (6MRC) determined by cryo-EM, no such asymmetry phenomenon was observed, and all subunits are in a similar conformation. The breakage of perfect seven-fold symmetry for intra-ring subunits was reported previously but to a milder degree. In the structures of apo GroEL and ATP-bound GroEL (PDB: 1OEL and 1KP8), the conformational variations of the apical domains relative to the equatorial domains among the intra-ring subunits were apparent, with spreads of 5° and 7° for apo and ATP-bound states, respectively^40,41^. Because no distinct gap between these two states was observed, it was realized the variations were likely induced by different packings due to different lattices (C222_1_ for 1OEL and P2_1_ for 1KP8). Similarly, the apical domain in the *cis*-ring of the bullet-shaped TtCpn60_14_Cpn10_7_ complex exhibited large deviation from the seven-fold symmetry^20^. The conformational variations of the apical domain in group 1 chaperonins are likely to be intrinsic to encapsulate different SPs rather than a single SP.

## Materials and methods

### Plasmid construction, protein expression, and purification

The full sequence of *Plasmodium falciparum* (isolate FCR-3/Gambia) mitochondria Cpn60 was retrieved from Uniprot entry number P34940 and then aligned to the crystal structure sequence of *E. coli* GroEL (PDB: 1AON). The flanking *N* and *C* terminal residues of *P. falciparum* Cpn60 were truncated to the alignment of *E. coli* GroEL. A D474A mutation was made in accordance to the ATP hydrolysis-deficient GroEL mutant (PDB: 3WVL)^45^ to inhibit ATP hydrolysis of Cpn60 and stabilize the complex for purification. Normal ATP binding is retained but ATP hydrolysis occurs at a 0.1% rate^14^. The mutant follows the same cycle as the wild-type but at a much slower rate, allowing for the observation of more transient intermediates. The overall structure of GroEL appears largely unaffected by the ATP hydrolysis-deficient mutation when compared to the wild-type football complex (PDB: 4PKO) with a rmsd value between the superimposed structures of 1.79 Å. The sequence was then synthesized by GenScript and cloned into a pET-28a vector with kanamycin antibiotic resistance genes. The sequence of mitochondria Cpn10 from *P. falciparum* was retrieved from Uniprot entry Q50JA6, synthesized by GenScript, and cloned into a pET-28a vector with the same antibiotic genes and a six-histidine tag. Expression of both constructs was accomplished by transformation of the plasmid into BL21 Rosetta (DE3) *E. coli* competent cells followed by initial culture growth of the transformed cells into a 50 mL flask with LB buffer and antibiotics. 20 mL of the initial culture was transferred into 1L LB flasks, incubated for 3 h at 37 °C, and induced with the addition of 1.0 mM isopropyl β-d-1-thiogalactopyranoside (IPTG). After overnight induction at 18 °C, the cell culture was spun down at 4000*g* for 15 min. The pellet was resuspended with 25 mL of buffer solution A containing 50 mM Tris pH 8.0, 100 mM NaCl, 10 mM KCl, and 10 mM MgCl_2_. A tablet of Pierce Protease inhibitor cocktail, 0.1 mM of PMSF, 0.1% (v/v) β-mercaptoethanol, and 5 mg of lysozyme was also added to the lysis buffer. Following sonication, the lysed cells were pelleted by centrifugation at 55,000*g* for 20 min. The supernatant of Cpn10 was purified using nickel-his affinity chromatography with two 10 mL washes containing buffer solution A and 10 mM imidazole. The protein was eluted from the nickel resin with 15 mL of buffer solution A containing 500 mM imidazole. The purified protein was concentrated to 1 mL using a 3 kDa cutoff Centricon and further purified with size-exclusion chromatography into buffer solution A. An average of 25 mg of Cpn10 protein was yielded from 1 L of culture. 1 mM of ATP was added to the supernatant of Cpn60 and incubated for an hour at 4 °C. 25 mg of purified Cpn10 was then mixed with the supernatant of Cpn60 and incubated for 1 h at 4 °C for complex formation. To purify the complex, a nickel-his pulldown was performed with the same procedure as the nickel-his affinity purification for Cpn10. The eluted complex was concentrated to 1 mL in a 10 kDa cutoff Centricon and further purified using size exclusion chromatography in buffer A. Fractions containing the complex were pooled and then concentrated to 20 mg/mL in a 100 kDa cutoff Centricon to remove unbound Cpn10 and used for crystallization.

### Negative stain electron microscopy

The sample quality and structural features were assessed by negative stain transmission electron microscopy in a Thermo Scientific Tecnai T20 microscope equipped with a charge-coupled device (CCD) camera. The protein samples (100 ng/mL) were applied to fresh plasma-cleaned carbon-coated grids (Quantifoil), followed by negative staining with 2% (w/v) uranyl acetate. Particles were auto-picked using Gautomatch (http://www.mrc-lmb.cam.ac.uk/kzhang/) and 2D classes were generated using RELION^49^.

### Crystallization and data collection

Initial screening of Cpn60 was performed using Qiagen's PEG Suite and JCSG Core Suite I–IV. 96-well screening trays were laid with a mosquito crystal robot with 70 µl of well buffer and a 1:1 ratio of 100 nL of protein to well buffer. After one week, crystals were produced in a condition containing 0.2 M lithium sulfate, 0.1 M phosphate citrate pH 4.2, and 10% (v/v) isopropanol. Optimization of conditions was then performed in 24-well hanging drop trays with a 1:1 ratio of 1 µl of protein to well buffer. After optimization of crystallization conditions 0.2 mm hexagonal prism shaped crystals grew in phosphate citrate buffer pH 4.4, 13% (v/v) isopropanol, and 0.21 mM of lithium sulfate. Crystals were cryoprotected with 30% (v/v) glycerol and then immediately submerged in liquid nitrogen for shipment to Argonne National Laboratory for data collection. X-ray diffraction data were collected at 100 K on beamline SERCAT 22-ID at the Argonne National Laboratory, Argonne, IL, USA through remote access mode. The data was recorded with an Eiger 16 M detector, processed, and scaled with the XDS package^50^. The data statistics are listed in Table 4.

### Structural solution and refinement

The structure of Cpn60mt-D474A was determined by molecular replacement with the program Phaser^51^ in Phenix package^34^ using the Cpn60 structure from *T. thermophilus* (PDB ID: 4V4O, TtCpn60) as the search model^20^. The structure was manually built using graphic package COOT^52^ and refined using Phenix package^53^. During refinement, sevenfold NCS torsional-angle restraints with three NCS groups consisting of the equatorial domain (residues 69–203 and 486–631), intermediate domain (residues 204–255 and 452–485), and apical domain (residues 256–451) were applied. Secondary structure restraints with Ramachandran restraints and planar peptide restraints were also applied. In the final cycle of refinement, the TLS refinement with three TLS groups (equatorial, intermediate, and apical domains) per chain, for a total of 21 TLS groups, was performed. The final R_work_ and R_free_ were 22.7% and 27.7%, respectively, with reasonably good geometry (Table 4). The atomic coordinates and structure factors have been deposited in the Protein Data Bank under accession number 7K3Z.

## Supplementary Information

Supplementary Information.

## Abbreviations

Cpn10: Chaperonin with molecular weight about 10 kDa
Cpn60: Chaperonin with molecular weight about 60 kDa
Hsp60: Heat shock protein with molecular weight of ~ 60 kDa
NCS: Non-crystallographic symmetry
SP: Substrate protein
