# Supplementary material for: Crystal structure of P. falciparum Cpn60 bound to ATP reveals an open dynamic conformation before substrate binding
Source: Sci Rep. 2021 Mar 15;11:5930. doi: 10.1038/s41598-021-85197-3 (PMC7960994; doi:10.1038/s41598-021-85197-3)
Supplement: Supplementary file 1 — Supplementary Information. [file 41598_2021_85197_MOESM1_ESM.docx]

**Supplementary information**

**Crystal structure of *P. falciparum* Cpn60 bound to ATP reveals an open dynamic conformation before substrate binding**

Brian Nguyen ^a^, Rui Ma ^a^, Wai Kwan Tang ^a^, Dashuang Shi ^a^ and Niraj Tolia ^a^

^a^ Laboratory of Malaria Immunology and Vaccinology, National Institute of Allergy and Infectious Diseases, National Institute of Health, Bethesda, Maryland, USA.

To whom correspondence should be addressed:

Dr. Niraj Tolia

Senior Investigator

Chief, Host-Pathogen Interactions and Structural Vaccinology Section

Laboratory of Malaria Immunology and Vaccinology

Division of Intramural Research

National Institute of Allergy and Infectious Disease

National Institutes of Health

Rm 4NN08, Building 29B, National Institutes of Health, 9000 Rockville Pike, Bethesda, Maryland 20892

e-mail: [niraj.tolia@nih.gov](mailto:niraj.tolia@nih.gov) Tel: 301-761-7537

**Table S1. Structures of chaperonin 60 in Protein Data Bank**

| PDB ID | Resolution | Methods | Organism | Composition | Ligand | Mutations | References |
| --- | --- | --- | --- | --- | --- | --- | --- |
| 4V4O | 2.8 | X-ray | T. thermophilus | Cpn60_14_Cpn10_7_ | ADP |  | [1] |
| 4PJ1 | 3.2 | X-ray | H. sapiens | Cpn60_14_Cpn10_14_ | ADP |  | [2] |
| 6MRC | 3.1 | EM | H. sapiens | Cpn60_14_Cpn10_14_ | ADP |  | [3] |
| 6MRD | 3.8 | EM | H. sapiens | Cpn60_7_Cpn10_7_ | ADP |  | [3] |
| 6HT7 | 3.7 | X-ray | H. sapiens | Cpn60_14_Cpn10_14_ | ADP+BeF_3_ |  | [3] |
| 1IOK | 3.2 | X-ray | P. denitrificans | Cpn60_14_ |  |  | [4] |
| 1PCQ | 2.8 | X-ray | E. coli | Cpn60_14_Cpn10_7_ | ADP+AlF_3_ |  | [5] |
| 1XCK | 2.9 | X-ray | E. coli | Cpn60_14_ |  |  | [6] |
| 1GRU | 12.5 | EM | E. coli | Cpn60_14_Cpn10_7_ | ADP+ATP |  | [7] |
| 2C7C | 7.7 | EM | E. coli | Cpn60_14_Cpn10_7_ | ATP |  | [8] |
| 2C7D | 8.7 | EM | E. coli | Cpn60_14_Cpn10_7_ | ADP |  | [8] |
| 2CGT | 8.2 | EM | E.coli | Cpn60_14_Cpn10_7_ | ADP+gp31 |  | [9] |
| 4PKN | 3.7 | X-ray | E.coli | Cpn60_14_Cpn10_14_ | ADP+BeF_3_ |  | [10] |
| 1MNF | 3.0 | X-ray | E. coli | Cpn60_14_ | peptide |  | [11] |
| 3ZQ1 | 15.9 | EM | E. coli | Cpn60_14_Cpn10_7_ | ADP | L43Py (Cpn60) | [12] |
| 3ZO0 | 9.2 | EM | E. coli | Cpn60_14_Cpn10_7_ | ADP | L43Py (Cpn60) | [12] |
| 3ZPZ | 8.9 | EM | E. coli | Cpn60_14_Cpn10_7_ | ADP | L43Py (Cpn60) | [12] |
| 3E76 | 3.9 | X-ray | E. coli | Cpn60_14_ | Ti^+^ |  | [13] |
| 3C9V | 4.7 | EM | E. coli | Cpn60_14_ |  |  | [14] |
| 1SVT | 2.8 | X-ray | E. coli | Cpn60_14_Cpn10_7_ | ADP+ AlF_3_ |  | [15] |
| 1SX4 | 3.0 | X-ray | E. coli | Cpn60_14_Cpn10_7_ | ADP |  | [15] |
| 2NWC | 3.0 | X-ray | E. coli | Cpn60_14_ |  |  | [16] |
| 1PF9 | 3.0 | X-ray | E. coli | Cpn60_14_Cpn10_7_ | ADP |  | [5] |
| 4HEL | 3.2 | X-ray | E. coli | Cpn60_14_ |  |  | unpublished |
| 2EU1 | 3.3 | X-ray | E. coli | Cpn60_14_ |  | E461K | [17] |
| 1AON | 3.0 | X-ray | E. coli | Cpn60_14_Cpn10_7_ | ADP |  | [18] |
| 4PKO | 3.8 | X-ray | E. coli | Cpn60_14_Cpn10_14_ | ADP+BeF_3_ |  | [10] |
| 5OPX | 3.6 | X-ray | E. coli | Cpn60_14_Cpn10_14_ | ADP+BeF_3_ | A109C | [19] |
| 5OPW | 3.2 | X-ray | E. coli | Cpn60_14_ |  | A109C | [19] |
| 4WSC | 3.0 | X-ray | E. coli | Cpn60_14_ |  | K105A | unpublished |
| 2C7E | 14.9 | EM | E. coli | Cpn60_14_ | ATP | D398A | [7] |
| 1GR5 | 7.9 | EM | E. coli | Cpn60_14_ |  |  | [7] |
| 1SX3 | 2.0 | X-ray | E. coli | Cpn60_14_ | AGS |  | [15] |
| 5W0S | 3.5 | EM | E. coli | Cpn60_14_ |  |  | [20] |
| 2YEY | 4.5 | EM | E. coli | Cpn60_14_ |  | E134K | [17] |
| 1SS8 | 2.7 | X-ray | E. coli | Cpn60_14_ |  |  |  |
| 4AAU | 8.5 | EM | E. coli | Cpn60_14_ | ATP |  | [21] |
| 4AAS | 8.5 | EM | E. coli | Cpn60_14_ | ATP |  | [21] |
| 4AAR | 8.5 | EM | E. coli | Cpn60_14_ | ATP |  | [21] |
| 4AAQ | 8.0 | EM | E. coli | Cpn60_14_ | ATP |  | [21] |
| 4AB2 | 8.5 | EM | E. coli | Cpn60_14_ | ATP |  | [21] |
| 4AB3 | 8.5 | EM | E. coli | Cpn60_14_ | ATP |  | [21] |
| 1GRL | 2.8 | X-ray | E. coli | Cpn60_14_ |  |  | [22] |
| 3CAU | 4.2 | EM | E. coli | Cpn60_14_ |  |  | [14] |
| 1KP8 | 2.0 | X-ray | E. coli | Cpn60_14_ | AGS |  | [23] |
| 1OEL | 2.8 | X-ray | E. coli | Cpn60_14_ |  |  | [24] |
| 4KI8 | 2.7 | X-ray | E. coli | Cpn60_14_ | ADP |  | [25] |
| 4WGL | 3.1 | X-ray | E. coli | Cpn60_14_ |  | D83A/R197A | unpublished |
| 4V43 | 3.5 | X-ray | E. coli | Cpn60_14_ |  |  | unpublished |
| 3WVL | 3.8 | X-ray | E. coli | Cpn60_14_Cpn10_14_ | ATP | D398A | [26] |
| 5DA8 | 3.0 | X-ray | C. tepidum | Cpn60_14_ |  |  | unpublished |
| 5CDI | 3.8 | X-ray | C. reinhardtii | Cpn60_14_ |  |  | [27] |

| **Table S2. Relative rotation angle (°) of apical and intermediate domains** | | |
| --- | --- | --- |
| PDB | Apical domain | Intermediate domain |
| This study | 0 | 0 |
| 1KP8 | -39 | -34 |
| 1OEL | -38 | -34 |
| 2C7E | -5 | -15 |
| 4AQQ | -17 | -6 |
| 1PCQ_cis_ | 92 | 12 |
| 1PCQ_trans_ | -42 | -33 |
| 5OPX | 85 | 12 |
| 1IOK | -36 | -29 |
| 5DA8 | -42 | -36 |
| 5CDI | -38 | -35 |
| 6MRC | 91 | 12 |

| **Table S3 Temperature factors for equatorial, intermediate and apical domains (Å^2^)** | | | |  |
| --- | --- | --- | --- | --- |
| Subunit | Equatorial | Intermediate | Apical |  |
| A | 126.7 | 139.8 | 143.9 |  |
| B | 130.1 | 161.2 | 231.4 |  |
| C | 132.3 | 159.3 | 269.8 |  |
| D | 133.9 | 161.5 | 184.3 |  |
| E | 139.5 | 160.3 | 193.0 |  |
| F | 137.1 | 171.6 | 263.5 |  |
| G | 127.5 | 136.8 | 161.2 |  |
| Fig. S1. Sequence alignment of *Plasmodium falciparum* Cpn60 (PfCpn60), with other Cpn60s from *Thermus thermophilus* (TtCpn60)*, Homo sapiens* (HsCpn60), *Escherichia coli* (EcGroEL), *Paracoccus denitrificans* (PdCpn60) and *Chlamydomonas reinhardtii (*CrCpn60), whose structures were known. The secondary structures of PfCpn60 are marked and labeled on the top of sequences with α-helices, 3_10_-helices and π-helices displayed as medium, small and large squiggles respectively, β-strands rendered as arrows, strict β-turns as **TT** letters and strict α-turns as **TTT**. The identical residues are highlighted as red. The partial conserved residues are highlighted in yellow. The figure was created using web server ESPript 3.0 [28]. | | | | |

| 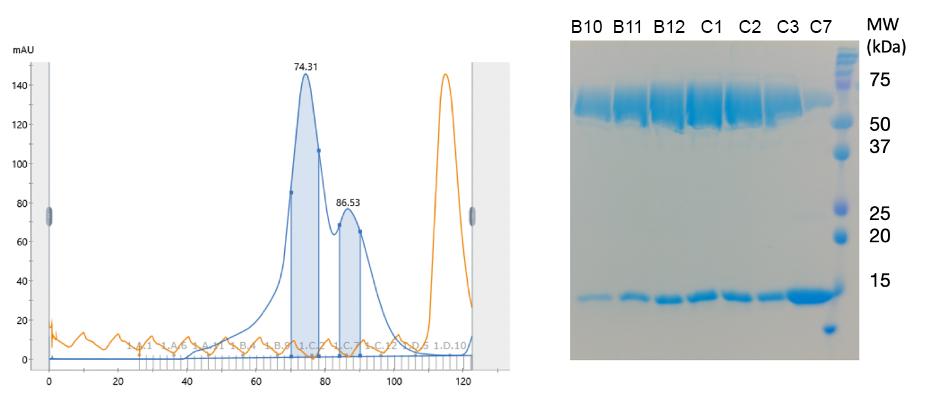  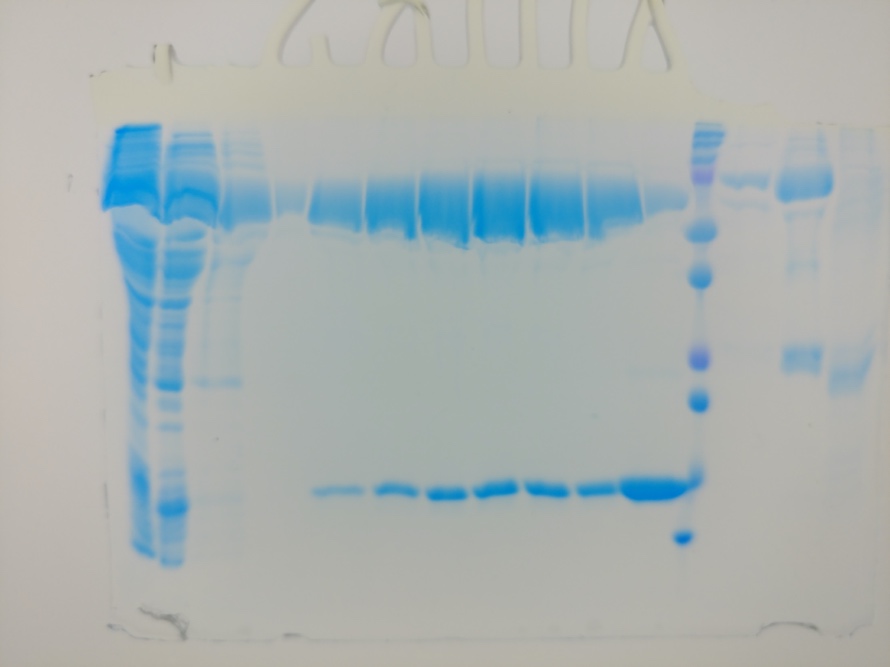 |
| --- |

**Fig. S2.** Chromatogram of size-exclusion chromatography on a Superose 6 HiLoad after copurification of Cpn60 D474A mutant with Cpn10 (left) and SDS-PAGE (right). The full-length gel is shown on the bottom.

**Fig. S3. Electron density around the extra 26-27 residues of PfCpn60.** The electron density is 2*Fo*-*Fc* map shown in 1.0σ in blue cage. The residues from K500 to S550 are shown in light-blue sticks.

|  |
| --- |

**Fig. S4. Electron density around double-ring interface of PfCpn60 and ATP.** (A) The electron density is 2*Fo*-*Fc* map shown in 1.0σ in blue cage around double-ring interface of PfCpn60. The molecular model is shown in sticks. (B) The electron density is a simulated annealing omitted *Fo*-*Fc* map shown at 3.0σ in green cage around ATP. The molecular models of ATP and surrounding residues are shown in sticks.

**Fig. S5. The ⍺3-⍺4 loop of PfCpn60.** (A) Structural comparison of the ⍺3-⍺4 loop of PfCpn60 with that of TtCpn60. (B) The electron density map (2*Fo*-*Fc*)(shown in 1.0σ in blue cage) around the ⍺3-⍺4 loop (from residue 137 to 167). The structures of PfCpn60 and TtCpn60 are shown in brown and light-blue ribbons, respectively. ATP and the side chains at ATP binding site are shown in sticks. The magnesium atom is shown as green sphere ball.

**References**

1. Shimamura, T., et al., *Crystal structure of the native chaperonin complex from Thermus thermophilus revealed unexpected asymmetry at the cis-cavity.* Structure, 2004. **12**(8): p. 1471-80.

2. Nisemblat, S., et al., *Crystal structure of the human mitochondrial chaperonin symmetrical football complex.* Proc Natl Acad Sci U S A, 2015. **112**(19): p. 6044-9.

3. Gomez-Llorente, Y., et al., *Structural basis for active single and double ring complexes in human mitochondrial Hsp60-Hsp10 chaperonin.* Nat Commun, 2020. **11**(1): p. 1916.

4. Fukami, T.A., et al., *Crystal structure of chaperonin-60 from Paracoccus denitrificans.* J Mol Biol, 2001. **312**(3): p. 501-9.

5. Chaudhry, C., et al., *Role of the gamma-phosphate of ATP in triggering protein folding by GroEL-GroES: function, structure and energetics.* EMBO J, 2003. **22**(19): p. 4877-87.

6. Bartolucci, C., et al., *Crystal structure of wild-type chaperonin GroEL.* J Mol Biol, 2005. **354**(4): p. 940-51.

7. Ranson, N.A., et al., *ATP-bound states of GroEL captured by cryo-electron microscopy.* Cell, 2001. **107**(7): p. 869-79.

8. Ranson, N.A., et al., *Allosteric signaling of ATP hydrolysis in GroEL-GroES complexes.* Nat Struct Mol Biol, 2006. **13**(2): p. 147-52.

9. Clare, D.K., et al., *An expanded protein folding cage in the GroEL-gp31 complex.* J Mol Biol, 2006. **358**(3): p. 905-11.

10. Fei, X., et al., *Formation and structures of GroEL:GroES2 chaperonin footballs, the protein-folding functional form.* Proc Natl Acad Sci U S A, 2014. **111**(35): p. 12775-80.

11. Wang, J. and L. Chen, *Domain motions in GroEL upon binding of an oligopeptide.* J Mol Biol, 2003. **334**(3): p. 489-99.

12. Chen, D.H., et al., *Visualizing GroEL/ES in the act of encapsulating a folding protein.* Cell, 2013. **153**(6): p. 1354-65.

13. Kiser, P.D., G.H. Lorimer, and K. Palczewski, *Use of thallium to identify monovalent cation binding sites in GroEL.* Acta Crystallogr Sect F Struct Biol Cryst Commun, 2009. **65**(Pt 10): p. 967-71.

14. Ludtke, S.J., et al., *De novo backbone trace of GroEL from single particle electron cryomicroscopy.* Structure, 2008. **16**(3): p. 441-8.

15. Chaudhry, C., et al., *Exploring the structural dynamics of the E.coli chaperonin GroEL using translation-libration-screw crystallographic refinement of intermediate states.* J Mol Biol, 2004. **342**(1): p. 229-45.

16. Kiser, P.D., D.T. Lodowski, and K. Palczewski, *Purification, crystallization and structure determination of native GroEL from Escherichia coli lacking bound potassium ions.* Acta Crystallogr Sect F Struct Biol Cryst Commun, 2007. **63**(Pt 6): p. 457-61.

17. Cabo-Bilbao, A., et al., *Crystal structure of the temperature-sensitive and allosteric-defective chaperonin GroELE461K.* J Struct Biol, 2006. **155**(3): p. 482-92.

18. Xu, Z., A.L. Horwich, and P.B. Sigler, *The crystal structure of the asymmetric GroEL-GroES-(ADP)7 chaperonin complex.* Nature, 1997. **388**(6644): p. 741-50.

19. Yan, X., et al., *GroEL Ring Separation and Exchange in the Chaperonin Reaction.* Cell, 2018. **172**(3): p. 605-617 e11.

20. Roh, S.H., et al., *Subunit conformational variation within individual GroEL oligomers resolved by Cryo-EM.* Proc Natl Acad Sci U S A, 2017. **114**(31): p. 8259-8264.

21. Clare, D.K., et al., *ATP-triggered conformational changes delineate substrate-binding and -folding mechanics of the GroEL chaperonin.* Cell, 2012. **149**(1): p. 113-23.

22. Braig, K., et al., *The crystal structure of the bacterial chaperonin GroEL at 2.8 A.* Nature, 1994. **371**(6498): p. 578-86.

23. Wang, J. and D.C. Boisvert, *Structural basis for GroEL-assisted protein folding from the crystal structure of (GroEL-KMgATP)14 at 2.0A resolution.* J Mol Biol, 2003. **327**(4): p. 843-55.

24. Braig, K., P.D. Adams, and A.T. Brunger, *Conformational variability in the refined structure of the chaperonin GroEL at 2.8 A resolution.* Nat Struct Biol, 1995. **2**(12): p. 1083-94.

25. Fei, X., et al., *Crystal structure of a GroEL-ADP complex in the relaxed allosteric state at 2.7 A resolution.* Proc Natl Acad Sci U S A, 2013. **110**(32): p. E2958-66.

26. Koike-Takeshita, A., et al., *Crystal structure of a symmetric football-shaped GroEL:GroES2-ATP14 complex determined at 3.8A reveals rearrangement between two GroEL rings.* J Mol Biol, 2014. **426**(21): p. 3634-41.

27. Zhang, S., et al., *Structural insight into the cooperation of chloroplast chaperonin subunits.* BMC Biol, 2016. **14**: p. 29.

28. Robert, X. and P. Gouet, *Deciphering key features in protein structures with the new ENDscript server.* Nucleic Acids Res, 2014. **42**(Web Server issue): p. W320-4.
